# Quality of life, care needs and priorities in atrial fibrillation: the impact of number and patterns of comorbidities

**DOI:** 10.1007/s11136-025-04117-4

**Published:** 2026-04-01

**Authors:** Caterina Trevisan, Adele Ravelli, José Miguel Rivera-Caravaca, Bruno Micael Zanforlini, Giuseppe Sergi, Gheorghe-Andrei Dan, Anca Rodica Dan, Vanessa Roldán, Francisco Marín Ortuño, Søren Paaske Johnsen, Mirko Petrovic, Davide Liborio Vetrano, Donato Giuseppe Leo, Deirdre A. Lane, the AFFIRMO consortium, Riccardo Proietti, Pia Cordsen, Gregory Lip, Deirdre Lane, Martin O’Flaherty, Carrol Gamble, Iain Buchan, Christodoulos Kypridemos, Brendan Collins, Donato Leo, Delphine De Smedt, Stefanie De Buyser, Cheima Amrouch, Amaia Calderón-Larrañaga, Lu Dai, Stefania Maggi, Marianna Noale, Elisabeta Badila, Nicola Ferri, Alessandra Buja, Vincenzo Stefano Rebba, Tatjana Potpara, Laura Vivani, Silvia Anastasia, Alessandro Ferri, Gehad Shehata, Nadia Rosso, Marco Cicerone, Jacek Marczyk, Trudie Lobban, Georg Ruppe, Federica Censi, Roberto Da Cas, Cecilia Damiano, Benedetta Marcozzi, Guendalina Graffigna, Caterina Bosio, Lorenzo Palamenghi, Serena Barello, Aldo Pietro Maggioni, Andrea Lorimer, Donata Lucci, Dipak Kalra, Nathan Lea, John Ainsworth, Charlotte Stockton-Powdrell, Alam Sanaullah, Francisco Marín Ortuño, Mariya Tokmakova

**Affiliations:** 1 Department of Medical Sciences, University of Ferrara, Ferrara, Italy; 2 Department of Medicine , University of Padua, Via Giustiniani 2, 35128, Padova, Italy; 3 Aging Research Center, Department of Neurobiology, Care Sciences and Society(NVS), Karolinska Institutet-Stockholm University, Solna, Sweden; 4 Faculty of Nursing, University of Murcia, Instituto Murciano de Investigación Biosanitaria Pascual Parrilla (IMIB-Pascual Parrilla), CIBERCV, Murcia, Spain; 5 Carol Davila University of Medicine and Pharmacy, Bucharest, Romania; 6 The Academy of Romanian Scientists (AOSR), Bucharest, Romania; 7 Cardiology Department, Colentina University Hospital, Bucharest, Romania; 8 Department of Hematology, Hospital Clínico Universitario Virgen de la Arrixaca, University of Murcia, Instituto Murciano de Investigación Biosanitaria Pascual Parrilla (IMIB-Pascual Parrilla), Murcia, Spain; 9 Department of Cardiology, Hospital Clínico Universitario Virgen de la Arrixaca, University of Murcia, Instituto Murciano de Investigación Biosanitaria Pascual Parrilla (IMIB-Pascual Parrilla), CIBERCV, Murcia, Spain; 10 Aalborg University Hospital, Aalborg, Denmark; 11 Danish Center for Health Services Research, Department of Clinical Medicine, Aalborg University, Aalborg, Denmark; 12 Section of Geriatrics, Department of Internal Medicine and Paediatrics, Ghent University, Ghent, Belgium; 13 Stockholm Gerontology Research Center, Stockholm, Sweden; 14 Liverpool Centre for Cardiovascular Sciences, University of Liverpool and Liverpool Heart and Chest Hospital, Liverpool, United Kingdom; 15 Department of Cardiovascular and Metabolic Medicine, Faculty of Health and Life Sciences, Institute of Life Course and Medical Sciences, University of Liverpool, Liverpool, United Kingdom

**Keywords:** Atrial fibrillation, Quality of life, Patient preference, Caregivers, Comorbidity patterns

## Abstract

**Purpose:**

Atrial fibrillation (AF) often coexists with multiple chronic conditions, worsening health-related quality of life (HRQoL) and increasing the burden on patients and caregivers. While multimorbidity is known to worsen clinical outcomes, the role of distinct comorbidity patterns in shaping patients’ experience remains unclear. This cross-sectional study assessed whether the number and patterns of comorbidities differentially affect HRQoL, care needs, and priorities of AF patients and caregivers.

**Methods:**

An online survey on living with AF and multimorbidity was disseminated between May 2022 and January 2023 in the UK, Italy, Spain, Romania, and Denmark. The analysis included 633 AF patients (46.9% females, median age 73 years) and 198 caregivers (26.8% females, median age 57 years). Exposure variables were the number and patterns (derived through latent class analysis) of comorbidities. Outcomes included HRQoL (measured with the EQ-5D-3L), perceived management problems and health priorities assessed through a structured questionnaire developed ad hoc for the survey.

**Results:**

Three patterns emerged: unspecific (65.5%), diabetes-kidney-liver (18.2%), and complex (16.4%). More comorbidities and belonging to the complex pattern were associated with worse HRQoL, mainly due to limited mobility, dependency, and pain. Main issues were managing multiple diseases, medi ions, and appointments. The diabetes-kidney-liver group prioritized improving quality of life (OR=3.08, 95%CI:1.68–6.00) and living longer (OR=1.67, 95%CI:1.05–2.64), while pain relief was a distinct priority in the complex pattern (OR=2.32, 95%CI:1.38–3.86).

**Conclusion:**

Both number and combinations of AF comorbidities shape patients’ and caregivers’ experiences. Considering comorbidity profiles can help define targeted care plans and caregiver support initiatives.

**Supplementary Information:**

The online version contains supplementary material available at 10.1007/s11136-025-04117-4.

## Introduction

Atrial fibrillation (AF), the most common arrhythmia in the older population [1], can substantially affect individual functionality and quality of life [2, 3], leading to a burden that falls both on patients and their caregivers [2, 4]. Previous studies have found that, even over short timeframes, around one in five patients with AF experience clinically meaningful worsening in health status and quality of life [5, 6].

In addition to the disease symptoms and clinical consequences, a relevant contribution to the burden of AF on quality of life and health status is attributable to comorbidities [3, 5–8]. The current literature shows that the presence of comorbidities affects the quality of life not only of AF patients [3, 5, 7] but also of their caregivers in relation to the time devoted to providing assistance and related burden [4]. In this context, some specific comorbidities such as cardiometabolic, musculoskeletal, and pulmonary diseases may affect quality of life more than others [3, 5, 9]. However, the extent each additional comorbidity can influence the quality of life and care needs of patients with AF and their caregivers is still unknown. Despite increasing evidence supports the importance of investigating multimorbidity not only quantitatively but also qualitatively [10], no studies so far have examined if quality of life, perceived care needs, and quality performance indicators of AF patients can vary as a function of their comorbidities’ pattern.

This topic is central since the coexistence of numerous comorbidities is most frequent among older AF patients [4, 11]. Such a high prevalence exists because many diseases are risk factors for AF development and because, in turn, AF can predispose to the onset of further acute and chronic conditions [11–13].

In line with the goals of the “Atrial Fibrillation Integrated Approach in Frail, Multimorbid, and Polymedicated Older People” (AFFIRMO) project, the identification of patients’ needs and priorities according to a holistic view that considers also their co-existing chronic conditions may help define personalized care pathways. Therefore, the present study aims to evaluate whether the number and patterns of comorbidities impacts quality of life, the main health-related problems and needs of AF patients and caregivers using real-world data from the AFFIRMO survey.

## Methods

### Study design and population

This cross-sectional study used data collected from an online survey as part of the AFFIRMO project. The main goal of this AFFIRMO task was to ascertain the experience of living with AF and multimorbidity and to identify the main health-related problems and needs of multimorbid patients with AF and caregivers. Inclusion criteria were age ≥18 years, having (or assisting a person with) at least one health condition in addition to AF, and ability to provide informed consent to participate in the survey. For the present analysis, of the 659 AF patients and 201 caregivers who participated in the survey, we included 633 (96.1%) patients and 198 (98.5%) caregivers with complete data on comorbidities, as these constituted our primary exposure measure.

The survey content was developed by a multidisciplinary team of physicians (geriatricians, cardiologists, internal medicine) and researchers experienced in AF, nurses, psychologists, and patient representatives, and the electronic platform was implemented by Advice Pharma Group srl. The survey was translated from English to each local language (Italian, Romanian, Spanish, and Danish) by professional native speakers, and included validated questionnaires (language appropriate where available). For each country, the referent persons for the study checked and approved the translated version of the survey (the English version of the survey is available along with a previous publication [14]).

The survey dissemination was performed by researchers involved in the project in local outpatient and inpatient clinics and via patients’ organisations from May 31, 2022, to January 31, 2023. The study protocol was approved by the Ethics committees from the United Kingdom (REC 21/YH/0307), Italy (015534, ref. 5308/AO/22), Spain (2021-12-15-HCUVA), and Romania (2SNI/13.01.2022). According to the guidelines of the Danish Research Ethics Committee, ethical approval was not required for Denmark. Before starting the online survey, each participant had to provide informed consent to be involved in the study by checking a box at the start of the online survey.

### Data collection

For the present study, we collected data on sociodemographic characteristics and the presence of comorbidities, from which we computed the total number of diseases and identified patterns of chronic conditions (see Statistical Analysis section). Health-related quality of life was assessed using the EQ-5D-3L questionnaire [15, 16], a standardized measure developed by the EuroQol Group to evaluate five domains, mobility, self-care, usual activities, pain/discomfort, and anxiety/depression, each rated at three levels of severity. The EQ-5D-3L also includes a visual analogue scale (EQ-VAS) ranging from 0 (worst imaginable health state) to 100 (best imaginable health state). All translated versions used were officially validated and cross-culturally adapted by the EuroQol Research Foundation. Moreover, a total EQ-5D-3L index was computed using validated tools and value sets [17]. Finally, the main challenges in living with or managing multimorbidity in AF patients and their caregivers, as well as key health outcomes, were evaluated based on a predefined set of alternatives developed by the project’s multidisciplinary team. A detailed overview of survey content is presented in Online Resources, Supplementary Table 1.

### Statistical analysis

Quantitative variables were expressed as mean (standard deviation, SD) or median (interquartile range, IQR), as appropriate, based normality of the distribution. Categorical variables were expressed as count and relative frequency (percentage).

A latent class analysis (LCA) was performed using the *poLCA package* in R to identify the patterns of diseases among patients who participated in the survey and patients assisted by caregivers. For this analysis, we considered diseases with a prevalence ≥2% in the study population (multiple sclerosis and Parkinson’s disease were excluded for this reason). The model with the best number of classes was detected by evaluating the Akaike Information Criterion (AIC) and the Bayesian Information Criterion (BIC), whose lower values correspond to better fitness. To characterise and label each latent class we considered two parameters: the observed/expected ratios of diseases (i.e. the ratio between the prevalence of a disease in a class and the prevalence of the disease in the study population) and disease exclusivity (i.e. the ratio between the number of disease cases in a class and the total number of cases of that disease in the study population), according to previous studies [18, 19]. Diseases with an observed/expected ratio ≥2 and an exclusivity ≥25% were considered to characterise a specific class.

The comparison of the main characteristics and HRQoL of participants belonging to different comorbidity classes was performed using the ANOVA or Kruskal-Wallis for quantitative variables, and Chi-squared test for the categorical variables.

The association between the total number of diseases and comorbidity classes with binary outcomes concerning HRQoL, main health-related problems, and important health outcomes was assessed by logistic regression analysis. The strength of these associations was expressed as *odds ratios* (OR) and 95% confidence intervals (95% CI). The association between the total number of diseases and comorbidity classes with the total score related to the EQ-5D-3L and EQ-VAS was tested through linear regression analysis. The strength of these associations was expressed as β coefficient and 95% CIs. Logistic and linear regression analyses were adjusted for potential confounders. For patients, analyses were adjusted for age, sex, educational level, and living arrangements. For caregivers, these included age, sex, educational level, time spent caregiving, and being a formal/informal caregiver.

All analyses were two-tailed, with a p-value <0.05 considered statistically significant, and were performed through the R software (version 4.3.3).

## Results

The characteristics of the 633 patients (46.9% males, median age 73 years) and 198 caregivers (26.8% males, median age 57 years) included are shown in Table 1. Of the patients involved, 52.9% were from the UK, 19% from Spain, 14.4% from Romania, 13.3% from Italy, and 0.5% from Denmark. Of the caregivers, 39.9% were from Romania, 32.8% from Spain, 24.2% from Italy, and 3% from the UK. Around 40% of patients and caregivers had a degree or a higher educational level. Most patients lived at home with family (62.9%) or alone (25.8%), while a minority were assisted part-time or full-time at home or in a nursing home. Most caregivers were informal (91.4%), and one in five provided full-time assistance. The median number of comorbidities of the AF patients who participated in the survey or were assisted by the involved caregivers was 3.

**Table 1:** Characteristics of patients and caregivers of patients with atrial fibrillation participating in the survey

	Overall	Comorbidity pattern			
		Unspecific	Diabetes-kidney-liver	Complex	p-value
*Patients*					
n	633 (100)	447 (70.6)	97 (15.3)	89 (14.1)	
Age (years)	73 [65, 78]	71 [64, 77]	75 [68, 79]	76 [69, 83]	<0.001
Male sex	297 (46.9)	196 (43.8)	58 (59.8)	43 (48.3)	0.016
Level of education			<0.001		
Primary	93 (14.7)	48 (10.7)	21 (21.6)	24 (27.0)	
Secondary	256 (40.4)	175 (39.1)	42 (43.3)	39 (43.8)	
Degree or above	265 (41.9)	211 (47.2)	32 (33.0)	22 (24.7)	
Living arrangement				<0.001	
At home alone	163 (25.8)	124 (27.7)	17 (17.5)	22 (24.7)	
At home with family	398 (62.9)	287 (64.2)	63 (64.9)	48 (53.9)	
At home with part-time assistance	50 (7.9)	28 (6.3)	9 (9.3)	13 (14.6)	
At home with full-time assistance or in NH	22 (3.5)	8 (1.8)	8 (8.2)	6 (6.7)	
Number of comorbidities	3 [2, 4]	2 [1, 3]	4 [3, 4]	6 [5, 7]	<0.001
*Caregivers*					
n	198 (100)	97 (49.0)	54 (27.3)	47 (23.7)	
Age (years)	57 [48, 71]	54 [47, 66]	58 [48, 70]	60 [52, 80]	0.042
Male sex	53 (26.8)	18 (18.6)	14 (25.9)	21 (44.7)	0.004
Level of education					0.093
Primary	25 (12.6)	7 (7.2)	7 (13.0)	11 (23.4)	
Secondary	75 (37.9)	36 (37.1)	22 (40.7)	17 (36.2)	
Degree or above	76 (38.4)	39 (40.2)	22 (40.7)	15 (31.9)	
Caregiving time					0.36
<6 h/day, not every day	100 (50.5)	55 (56.7)	23 (42.6)	22 (46.8)	
<6 h/day, every day	56 (28.3)	25 (25.8)	19 (35.2)	12 (25.5)	
Full-time	42 (21.2)	17 (17.5)	12 (22.2)	13 (27.7)	
Formal caregiver	17 (8.6)	11 (11.3)	3 (5.6)	3 (6.4)	0.395
Number of comorbidities (of the assisted person)	3 [2, 5]	2 [2, 3]	4 [3, 4]	7 [6, 7.5]	<0.001

Numbers are median [interquartile range] or number (%), as appropriate. Data on educational level were unavailable for 19 patients and 22 caregivers. For caregivers, comorbidity patterns refer to their assisted person. *Abbreviations:* NH, nursing home.

**Fig. 1:**
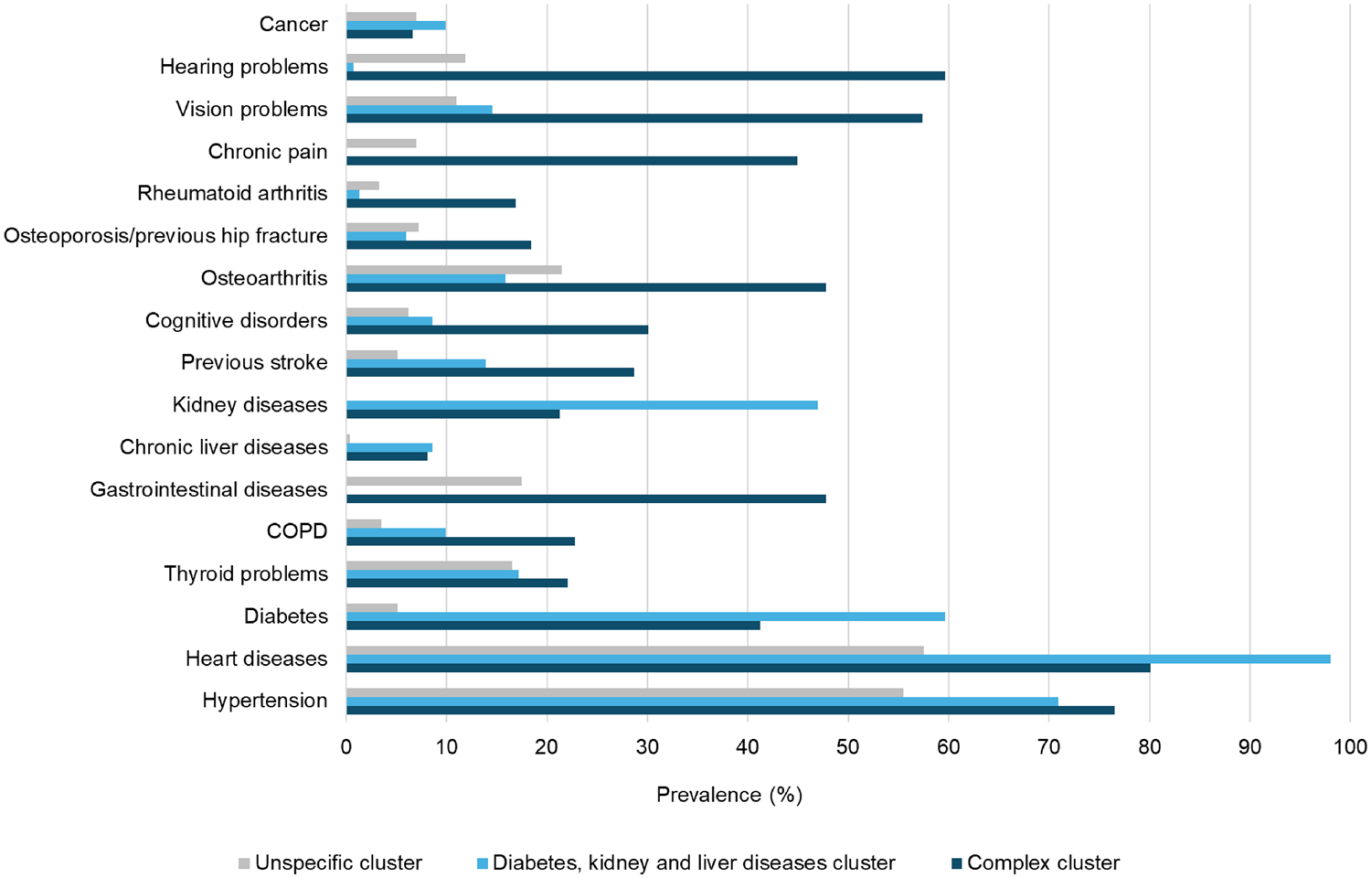
Prevalence of chronic conditions in the patterns identified through the latent class analysis

The LCA identified three patterns of comorbidities (Figure 1; Online Resources, Supplementary Table 2). The first pattern included 70.6% of the patients directly involved in the survey and 49% of the patients assisted by the participating caregivers and did not show any main characterising disease (*unspecific pattern*). The second pattern included 15.3% of patients and 27.3% of the caregiver-assisted patients, and was characterised by diabetes, chronic kidney and liver diseases (*Diabetes-kidney-liver pattern*); while, in the third pattern, including 14.1% of patients and 23.7% of the caregiver-assisted patients, multiple chronic disorders co-existed, especially COPD, gastrointestinal, rheumatologic and neurological diseases, cognitive disorders, chronic pain and sensory deficits (*complex pattern*). When comparing the three patterns, the complex patterns included older patients with lower education and higher assistance requirements compared with the other two patterns. Caregivers of patients belonging to the complex pattern were slightly older and more likely to be males than the other patterns; they did not differ in time spent caregiving and frequency of formal caregivers. The median number of comorbidities was two in the unspecific, four in the diabetes-kidney-liver, and six to seven in the complex pattern (Table 1).

### Quality of life

Both patients and caregivers of patients with a complex pattern of comorbidities were more likely to have problems in mobility, self-care, and usual activities and worse health status assessed through the VAS; patients with a complex pattern showed also lower total EQ-5D-3L score (Online Resources, Supplementary Table 2). Moreover, a significantly higher frequency of problems with pain or discomfort emerged among patients (but not caregivers) in the complex pattern. These results were confirmed in logistic and linear regression analyses (Table 2; Online Resources, Supplementary Tables 3 and 4). After adjusting for potential confounders, the complex pattern was associated with a two- to four-fold higher probability of presenting problems in mobility, self-care, and usual activities than the unspecific pattern. Among patients, those in the diabetes-kidney-liver pattern were more likely to report problems in self-care (OR=2.80, 95%CI: 1.59 – 4.91) whereas those in the complex pattern reported more pain/discomfort (OR=1.83, 95%CI: 1.10–3.12). Considering the number of comorbidities (Table 2), with each additional disease, the probability of patients reporting problems increased by 35% for mobility, 42% with self-care, 33% with usual activities, 28% with pain/discomfort, and 19% with anxiety/depression. Moreover, a significant association was found between the number of comorbidities of the assisted patient and the probability that caregivers had mobility problems.

**Table 2:** Binary logistic regression for the association between the number and pattern of comorbidities and quality of life of patients and caregivers of patients with atrial fibrillation

EQ-5D domains	Odds ratio (95% Confidence Interval)		
	N. comorbidities *(per each 1-disease increase)*	Comorbidity pattern*	
		Diabetes-kidney-liver	Complex
*Mobility*			
Patients	1.35 (1.21–1.52) *p*<0.001	1.03 (0.63–1.66) *p*=0.912	2.69 (1.59–4.67) *p*<0.001
Caregivers	1.23 (1.03–1.46) *p*=0.02	0.79 (0.29–2.10) *p*=0.648	3.60 (1.42–9.35) *p*=0.007
*Self-care*			
Patients	1.42 (1.26–1.62) *p*<0.001	2.80 (1.59–4.91) *p*<0.001	3.28 (1.86–5.76) *p*<0.001
Caregivers	1.22 (0.97–1.55) *p*=0.087	0.42 (0.08–1.78) *p*=0.261	4.03 (1.10–15.68) *p*=0.037
*Usual activities*			
Patients	1.33 (1.19–1.49) *p*<0.001	0.82 (0.50–1.32) *p*=0.413	2.19 (1.30–3.77) *p*=0.004
Caregivers	1.16 (0.98–1.37) *p*=0.09	0.81 (0.29–2.14) *p*=0.678	3.51 (1.39–9.11) *p*=0.009
*Pain/discomfort*			
Patients	1.28 (1.15–1.43) *p*<0.001	0.65 (0.41–1.04) *p*=0.072	1.83 (1.10–3.12) *p*=0.023
Caregivers	0.95 (0.82–1.09) *p*=0.467	1.12 (0.55–2.30) *p*=0.752	0.91 (0.41–2.00) *p*=0.811
*Mental*			
Patients	1.19 (1.07–1.32) *p*=0.001	0.96 (0.60–1.53) *p*=0.859	1.52 (0.93–2.49) *p*=0.098
Caregivers	1.07 (0.92–1.24) *p*=0.389	0.71 (0.33–1.50) *p*=0.37	1.36 (0.60–3.11) *p*=0.462

^*^Reference category: Unspecific pattern. *Notes.* Models for the analysis on caregivers are adjusted for age, sex, education, caregiving time, formal/informal caregiver. Models for the analysis on patients are adjusted for age, sex, education, and living arrangements.

### Perceived main health-related problems

Concerning the main health problems (Table 3), the complex pattern was associated with higher odds of patients reporting problems related to mobility or need for help (OR=2.05, 95%CI: 1.21-3.42), compared with the unspecific pattern. In addition, patients with a complex comorbidity pattern seemed to be more likely to report issues with the number or logistics of medical appointments (OR = 1.79, 95% CI: 1.00–3.15) and insufficient financial resources (OR=2.27, 95%CI: 1.00-4.99), although these associations were not significant. Both patients in the diabetes-kidney-liver and the complex pattern had more than three-fold higher probability of reporting problems linked to the management of too many medications or diseases. Conversely, patients in the diabetes-kidney-liver pattern and caregivers assisting patients from the complex pattern of comorbidities were less likely to report communication issues with the doctor. Moreover, these caregivers, compared to the unspecific group, were more likely to report problems due to the management of their own diseases (OR=5.65, 95%CI: 1.86-18.95) and the responsibility of caring (OR=3.47, 95%CI: 1.07-11.68). In general, the higher the number of the patient’s comorbidities, the greater the probability of reporting problems concerning medical appointments, management of multiple medications or diseases (both for patients and caregivers), and mobility or assistance needs.

**Table 3:** Association between the number and pattern of comorbidities and main health problems of patients and caregivers of patients with atrial fibrillation

Problems	Odds ratio (95% Confidence Interval)		
	N. comorbidities *(per each 1-disease increase)*	Comorbidity pattern*	
		Diabetes-kidney-liver	Complex
*Number of health appointments and logistics*			
Patients	1.16 (1.02–1.31) *p*=0.02	1.41 (0.77–2.49) *p*=0.248	1.79 (1.00–3.15) *p*=0.047
Caregivers	0.99 (0.85–1.14) *p*=0.863	1.4 (0.67–2.92) *p*=0.37	0.74 (0.31–1.71) *p*=0.494
*Contacting/seeing a medical doctor*			
Patients	1.01 (0.91–1.12) *p*=0.862	0.93 (0.58–1.49) *p*=0.76	0.9 (0.54–1.47) *p*=0.665
Caregivers	1.05 (0.90–1.22) *p*=0.507	1.53 (0.67–3.48) *p*=0.307	1.61 (0.67–3.81) *p*=0.283
*Too many medications to take/health problems*			
Patients	1.43 (1.28–1.60) *p*<0.001	3.97 (2.45–6.57) *p*<0.001	3.34 (2.04–5.59) *p*<0.001
Caregivers	1.17 (1.02–1.34) *p*=0.03	1.5 (0.74–3.05) *p*=0.265	1.59 (0.74–3.45) *p*=0.236
*Mobility/assistance needs*			
Patients	1.27 (1.14–1.43) *p*<0.001	0.56 (0.28–1.03) *p*=0.072	2.05 (1.21–3.42) *p*=0.007
Caregivers	1.17 (0.97–1.41) *p*=0.085	1.6 (0.54–4.68) *p*=0.384	2.64 (0.89–7.85) *p*=0.076
Communicating with the doctor			
Patients	0.93 (0.82–1.05) *p*=0.229	0.38 (0.18–0.71) *p*=0.005	0.64 (0.34–1.15) *p*=0.149
Caregivers	0.66 (0.46–0.89) *p*=0.011	0.46 (0.12–1.41) *p*=0.203	0.11 (0.01–0.64) *p*=0.044
Anxiety/worry about patient’s health			
Patients	0.91 (0.82–1.02) *p*=0.10	0.92 (0.56–1.48) *p*=0.725	0.74 (0.43–1.23) *p*=0.252
Caregivers	1.00 (0.87–1.15) *p*=0.988	0.67 (0.31–1.40) *p*=0.297	0.9 (0.41–1.95) *p*=0.794
*Not having enough financial resources*			
Patients	1.15 (0.96–1.37) *p*=0.122	1.45 (0.58–3.32) *p*=0.401	2.27 (1.00–4.99) *p*=0.047
Caregivers	1.13 (0.95–1.35) *p*=0.159	1.54 (0.54–4.22) *p*=0.406	2.95 (1.07–8.17) *p*=0.035
*Anxiety/worry about own (caregiver) health*			
Caregivers	0.96 (0.76–1.20) *p*=0.741	0.69 (0.22–2.05) *p*=0.52	0.33 (0.08–1.22) *p*=0.119
*Managing own (caregiver) health problems*			
Caregivers	1.38 (1.14–1.68) *p*=0.001	2.49 (0.77–8.47) *p*=0.13	5.65 (1.86–18.95) *p*=0.003
*Responsibility of caring*			
Caregivers	1.19 (0.95–1.46) *p*=0.112	0.75 (0.15–2.94) *p*=0.692	3.47 (1.07–11.68) *p*=0.038

^*^Reference category: Unspecific pattern. *Notes.* Models for the analysis on caregivers are adjusted for age, sex, education, caregiving time, formal/informal caregiver. Models for the analysis on patients are adjusted for age, sex, education, and living arrangements.

### Important health outcomes

Compared with the unspecific comorbidity group, patients in the diabetes-kidney-liver pattern were more likely to report better HRQoL (OR=3.08, 95%CI: 1.68-6.00) and living longer (OR=1.67, 95%CI: 1.05-2.64) as important outcomes to them, while they gave less relevance to the maintenance of social and leisure activities (Table 4). Only pain reduction/relief was reported significantly more frequently by the complex pattern (OR=2.32, 95%CI: 1.38-3.86). Considering the number of comorbidities, for each additional disease the odds of reporting pain reduction/relief as an essential outcome increased, while the importance of maintaining social and leisure activities decreased.

**Table 4:** Binary logistic regression for the association between the number and pattern of comorbidities and health outcomes reported by patients and caregivers of patients with atrial fibrillation

Health outcomes	Odds ratio (95% Confidence Interval)		
	N. comorbidities *(per each 1-disease increase)*	Comorbidity pattern*	
		Diabetes-kidney-liver	Complex
Patients	1.03 (0.92–1.15) *p*=0.58	3.08 (1.68–6.00) *p*<0.001	1.03 (0.62–1.74) *p*=0.909
Caregivers	0.95 (0.82–1.11) *p*=0.503	0.85 (0.39–1.88) *p*=0.677	0.88 (0.38–2.10) *p*=0.765
Patients	0.92 (0.83–1.02) *p*=0.11	1.67 (1.05–2.64) *p*=0.029	0.66 (0.38–1.10) *p*=0.117
Caregivers	1.09 (0.93–1.27) *p*=0.284	0.62 (0.26–1.44) *p*=0.276	1.58 (0.67–3.72) *p*=0.294
Patients	1.28 (1.14–1.44) *p*<0.001	0.6 (0.31–1.10) *p*=0.114	2.32 (1.38–3.86) *p*=0.001
Caregivers	1.21 (1.04–1.41) *p*=0.013	0.92 (0.41–2.03) *p*=0.841	1.52 (0.66–3.49) *p*=0.319
Patients	1.03 (0.93–1.14) *p*=0.584	1.11 (0.69–1.80) *p*=0.67	0.99 (0.60–1.62) *p*=0.952
Caregivers	0.94 (0.82–1.08) *p*=0.378	1.4 (0.68–2.91) *p*=0.366	1.24 (0.57–2.74) *p*=0.592
Patients	0.93 (0.82–1.05) *p*=0.26	0.68 (0.36–1.21) *p*=0.203	0.79 (0.42–1.42) *p*=0.446
Caregivers	1.14 (0.95–1.36) *p*=0.144	0.94 (0.33–2.51) *p*=0.911	1.44 (0.51–3.89) *p*=0.475
Patients	0.84 (0.74–0.96) *p*=0.008	0.32 (0.15–0.60) *p*=0.001	0.66 (0.35–1.19) *p*=0.175
Caregivers	1.06 (0.86–1.28) *p*=0.584	1.64 (0.56–4.72) *p*=0.355	1.32 (0.39–4.12) *p*=0.643
Patients	1.13 (0.99–1.27) *p*=0.061	0.54 (0.24–1.08) *p*=0.101	1.54 (0.85–2.73) *p*=0.144
Caregivers	0.9 (0.74–1.07) *p*=0.248	0.8 (0.34–1.84) *p*=0.611	0.42 (0.14–1.13) *p*=0.102

^*^Reference category: Unspecific pattern. *Notes.* Models for the analysis on caregivers are adjusted for age, sex, education, caregiving time, formal/informal caregiver. Models for the analysis on patients are adjusted for age, sex, education, and living arrangements.

## Discussion

This study shows that both the number and patterns of comorbidities of AF patients significantly influence the quality of life, health-related problems, and primary outcomes that they and their caregivers perceive. Patients more comorbidities and greater clinical complexity had worse HRQoL, mostly due to limited mobility, dependency on daily activities, and pain or discomfort. Managing multiple diseases, medications, and health appointments were the main issues reported by these patients, with a substantial burden also on caregivers.

The three main comorbidities patterns found in this study partly align with previous findings, although larger cohorts identified more patterns due greater heterogeneity and statistical power [18, 20, 21]. Consistent with previous results [18, 20], most patients exhibited an unspecific comorbidity pattern, maintaining a relatively healthy clinical and functional status despite presenting a non-negligible prevalence of some cardiovascular diseases. The second pattern was characterized by diabetes, chronic kidney, and liver diseases. This pattern included patients requiring assistance in daily activities, probably due to their cardiometabolic conditions. Finally, around one in 10 patients presented with a complex pattern with multiple chronic conditions, including cardiovascular diseases, cognitive deficits, chronic pain, osteoarthritis, and sensory deficits. This frequency increased to almost one quarter when considering caregivers reports. Previous studies in broader cohorts of older adults with AF also identified a complex comorbidity pattern associated with worse health-related outcomes, including mortality and adverse cardiovascular events [18, 20]. However, to our knowledge, no studies have examined the relationship between comorbidity patterns and other relevant endpoints, such as HRQoL, perceived health-related problems, and outcomes to prioritize.

Regarding HRQoL, we found a significant impact of the number and pattern of comorbidities in patients and their caregivers. Each additional comorbidity affected patients’ quality of life across all domains. Among caregivers, the burden was mainly related to general health perception and mobility. The worsening of quality of life as a function of the number of comorbidities has been observed in previous studies of AF patients [3, 6, 7, 22]. Instead, concerning caregivers, only one survey investigated the burden of people assisting AF patients and found similar results [4]. Among comorbidity patterns, the greatest burden on HRQoL was observed for the complex pattern, which affected mobility, self-care, and daily activities in both patients and caregivers, as well as pain/discomfort only among the formers. Instead, the impact of the diabetes-kidney-liver pattern on HRQoL fell mostly on self-care. These findings complement previous observations, although those studies focused on single chronic diseases and not on their patterns [3, 5, 9, 22]. In particular, a heavier burden on health status and quality of life was previously demonstrated for cardiovascular comorbidities, diabetes, chronic kidney failure, musculoskeletal diseases, cancer, and psychocognitive disorders, with some sex differences [3, 5, 9, 22]. In AF patients, these diseases may further increase the need for medical visits and chronic treatments and could substantially impair individual functional status [23]. In addition, the management of these comorbidities in AF patients might be challenging due to possible interactions between medications related to AF (e.g., anticoagulants, antiarrhythmics) and those for comorbidities (e.g., anti-inflammatory drugs, painkillers) [24, 25], which may influence the onset of AF complications [26].

Accordingly, we found that the main challenges for those with more comorbidities and their caregivers, especially in the complex comorbidity pattern, were managing too many conditions, medications, and health appointments and having mobility difficulties. Only few surveys or qualitative studies so far provided comparative data to our findings on this matter [2, 27, 28]. In these works, AF patients reported a need for person-centred information on their health conditions and lifestyle, with a focus on the quality of life and the promotion of regular communications with physicians [2, 27, 28]. Additional problems were mostly related to physical health status, including fatigue management, weight control, sleep problems, and concern about AF complications [28]. Similarly, a survey of over five hundred caregivers from the UK, Italy, and Germany reported that the most common problems were in daily activities and mental and physical health dimensions4; however, each specific problem was not explored as a function of the patient comorbidities. That study also assessed caregiver burden, which increased when the assisted person had osteoarticular disorders, diabetes, or cancer [4]. These results are consistent with another study reporting a higher burden in caregivers of AF patients with frailty, disability, or health status deterioration [29].

Finally, it is worth noting that the number and patterns of patient comorbidities conditioned their perceived priorities. For individuals in the diabetes-kidney-liver pattern, important health outcomes were improving quality of life and living longer; instead, with the increasing number of comorbidities and in clinically complex patients, the exclusive priority was pain reduction and relief. These findings should encourage physicians to wonder routinely about key questions [30], such as: *Have the primary health needs and preferences been adequately discussed with the patient and caregiver? What are the realistic goals of the proposed/ongoing therapeutic intervention? Should palliative care be considered for symptom relief and improving quality of life?*

Among the study’s limitations and strengths, we recognize that the modest number of caregivers who participated in the survey could have affected the statistical power of our analyses. On the other hand, evaluating both patient- and caregiver-related outcomes is a strength of our work, considering the scarcity of data on these issues in the literature. Moreover, we did not always involve dyads of patients and caregivers. However, this allowed us to improve the representativeness of our sample since the more complex and frailest patients are less likely to participate directly in a survey; still, their caregivers could give voice to their experience.

Another limitation concerns missing data. Participants with incomplete information on comorbidities (our primary exposure) were excluded a priori from the analyses. Therefore, analyses were performed on a complete-case basis. Additionally, the educational level was unavailable for 19 patients and 22 caregivers. Missing data on comorbidities are likely missing at random, probably due to advanced age or cognitive impairment, making it difficult for participants to recall their complete medical history. In contrast, missing information on education is more likely to be not at random, as individuals with lower educational attainment may be less willing to report it. These patterns of missingness should be considered when interpreting the results.

Moreover, given the relatively small number of participants in some comorbidity classes, the study might have been underpowered to detect associations with certain outcomes (e.g., financial resources, number of medical appointments), and caution in their interpretation is warranted.

Another issue to be considered is the potential cultural differences across countries in the perceived care needs or health-related outcomes, as well as in the caregiving experience, which might have influenced our findings. Finally, as mentioned above, the results of the LCA could have been affected by the sample size of our study. Previous analyses undertaken in larger populations, especially when using data from national registers, found more comorbidity patterns, probably due to a greater heterogeneity of chronic diseases [18].

In conclusion, our study suggests that both the quantity and pattern of comorbidities may influence the quality of life, health-related needs, and priorities of AF patients and their caregivers. Applying a comprehensive evaluation of AF comorbidities and their impact on older adults could help better define care pathways with personalized goals based on patient’s preferences. Moreover, targeted initiatives to support caregivers−especially those assisting patients with complex comorbidity patterns−by providing personal and material resources must be prioritized in our healthcare and social system.

## Supplementary Information

Below is the link to the electronic supplementary material.

Supplementary file1

## Data Availability

The datasets generated during and/or analyzed during the current study are not publicly available due privacy reasons but are available from the corresponding author on reasonable request.
