# Supplementary material for: Quality of life, care needs and priorities in atrial fibrillation: the impact of number and patterns of comorbidities
Source: Qual Life Res. 2026 Apr 1;35(5):109. doi: 10.1007/s11136-025-04117-4 (PMC13043515; doi:10.1007/s11136-025-04117-4)
Supplement: Supplementary file 1 — Supplementary file1 [file 11136_2025_4117_MOESM1_ESM.docx]

**Supplementary Information: Online Resources**

**Quality of Life Research**

**Quality of life, care needs and priorities in atrial fibrillation:**

**the impact of number and patterns of comorbidities**

Caterina Trevisan, MD, PhD, Adele Ravelli, MS, José Miguel Rivera-Caravaca, RN, PhD, Bruno Micael Zanforlini, MD, Giuseppe Sergi, MD, PhD, Gheorghe-Andrei Dan, MD, PhD, Anca Rodica Dan, MD, Vanessa Roldán, MD, PhD, Francisco Marin Ortuño, MD, PhD, Søren Paaske Johnsen, MD, PhD, Mirko Petrovic, MD, PhD, Davide Liborio Vetrano, MD, PhD, Donato Giuseppe Leo, PhD, Deirdre A Lane, PhD on behalf of the AFFIRMO consortium

**Corresponding author**

Adele Ravelli

Department of Medicine, University of Padova

Via Giustiniani 2, 35128, Padova, Italy

Email: adele.ravelli@phd.unipd.it

**Supplementary Table 1. Main information collected in the AFFIRMO survey**

| Main areas of interest investigated | Collected information |
| --- | --- |
| Sociodemographic data | Age, sex, country, educational level (categorized as primary, secondary, and degree level or above), and living arrangements (categorised as living at home either alone, with family, with part-time to full-time assistance, or living in a nursing home). For caregivers, we assessed whether they were formal/informal, and the amount of time spent providing assistance. |
| Medical data (comorbidities) | Presence of the following diseases: arterial hypertension, heart diseases, diabetes mellitus, thyroid problems, chronic obstructive pulmonary disease (COPD), gastrointestinal diseases, chronic liver or kidney diseases, previous stroke, Parkinson disease, multiple sclerosis, cognitive disorders, osteoarthritis, osteoporosis/previous hip fracture, rheumatoid arthritis, chronic pain, oncologic diseases, and vision and hearing problems. |
| Health-related quality of life (HRQoL) | The EQ-5D-3L questionnaire^15^ assesses the presence of problems in five domains: mobility, self-care, usual activities, pain/discomfort, and anxiety/depression. For each domain, participants were asked to report whether they had no problems, some problems, or extreme problems. From these responses, an overall score of health-related quality of life was computed based on the current recommendations^16^. The survey also included the EQ Visual Analogue Scale (VAS), where participants had to indicate their perceived health status on a scale ranging from 0 (worst health) to 100 (best health). |
| Main problems in living or managing multimorbidity in AF patients and caregivers | Participants could indicate a maximum of three main problems from: having a high number of medical appointments, travel to medical appointments, having too many medications and/or health conditions to manage, insufficient financial resources, difficulties in contacting/seeing a medical doctor, anxiety/worry for own health, not having anyone to help, mobility problems, not understanding medical recommendations clearly, having problems contacting the doctor/healthcare team, and not having the opportunity to talk freely with the doctor about doubts and fears of health conditions and treatment. For caregivers only, problems also included: having their own health problems, anxiety/worry for the health of the assisted person, and responsibility of caring for someone else. Due to possible overlapping issues, some problems were grouped together for the analyses: too many medications to take/health problems (from “too many medications to manage” and “too many health problems”), number of health appointments and logistics (from “high number of medical appointments” and “travel to medical appointments”), contacting/seeing a medical doctor (“difficulties in contacting/seeing a medical doctor” and “having problems getting in contact with the doctor/healthcare team”), mobility/assistance needs (from “not having anyone else to help” and “mobility problems”), and communicating with the doctor (from “do not understand medical recommendations clearly” and “not having the opportunity to talk freely with the doctor about doubts and fears of health conditions and treatment”). |
| Health outcomes of importance | Participants could indicate a maximum of three health outcomes from: improvement of quality of life, maintenance of independence in daily life, living longer, pain reduction/relief, having less need for health care, maintenance of social and leisure activities, improvement of mental/emotional health, and being able to work. For the analyses, “maintenance of independence in daily life” and “being able to work” were grouped together since they can overlap as both include independence and maintenance of functional status. |

**Supplementary Table 2. Frequency of chronic diseases by comorbidity pattern**

|  |  | **Comorbidity pattern** | | |  |
| --- | --- | --- | --- | --- | --- |
|  | **Overall** | **Unspecific** | **Diabetes-kidney-liver** | **Complex** | **p-value** |
| n | 633 | 328 | 199 | 106 |  |
| High blood pressure | 389 (61.5) | 180 (54.9) | 138 (69.3) | 71 (67.0) | 0.002 |
| Heart disease | 399 (63.0) | 159 (48.5) | 167 (83.9) | 73 (68.9) | <0.001 |
| Diabetes | 108 (17.1) | 6 (1.8) | 69 (34.7) | 33 (31.1) | <0.001 |
| Thyroid problems | 108 (17.1) | 70 (21.3) | 23 (11.6) | 15 (14.2) | 0.01 |
| COPD | 41 (6.5) | 1 (0.3) | 24 (12.1) | 16 (15.1) | <0.001 |
| Gastrointestinal diseases | 128 (20.2) | 71 (21.6) | 7 (3.5) | 50 (47.2) | <0.001 |
| Chronic liver disease | 19 (3.0) | 8 (2.4) | 7 (3.5) | 4 (3.8) | 0.685 |
| Kidney disease | 62 (9.8) | 0 (0.0) | 42 (21.1) | 20 (18.9) | <0.001 |
| Previous stroke | 57 (9.0) | 13 (4.0) | 21 (10.6) | 23 (21.7) | <0.001 |
| Parkinson’s disease | 8 (1.3) | 5 (1.5) | 0 (0.0) | 3 (2.8) | 0.09 |
| Multiple sclerosis | 3 (0.5) | 2 (0.6) | 0 (0.0) | 1 (0.9) | 0.456 |
| Cognitive disorders | 48 (7.6) | 16 (4.9) | 4 (2.0) | 28 (26.4) | <0.001 |
| Osteoarthritis | 165 (26.1) | 95 (29.0) | 21 (10.6) | 49 (46.2) | <0.001 |
| Osteoporosis/previous hip fracture | 51 (8.1) | 32 (9.8) | 2 (1.0) | 17 (16.0) | <0.001 |
| Rheumatoid arthritis | 29 (4.6) | 14 (4.3) | 1 (0.5) | 14 (13.2) | <0.001 |
| Chronic pain | 77 (12.2) | 30 (9.1) | 0 (0.0) | 47 (44.3) | <0.001 |
| Vision problems | 119 (18.8) | 27 (8.2) | 34 (17.1) | 58 (54.7) | <0.001 |
| Hearing problems | 105 (16.6) | 35 (10.7) | 13 (6.5) | 57 (53.8) | <0.001 |
| Cancer | 40 (6.3) | 27 (8.2) | 7 (3.5) | 6 (5.7) | 0.093 |

*Abbreviations*: COPD, chronic obstructive pulmonary disease.

**Supplementary Table 3. Quality of life of patients and caregivers of patients with atrial fibrillation participating in the survey (total sample and by comorbidity pattern)**

|  | **Patients** | | | |  | **Caregivers** | | | |  |
| --- | --- | --- | --- | --- | --- | --- | --- | --- | --- | --- |
|  | **All** | **Unspecific**  **pattern** | **DKL diseases pattern** | **Complex pattern** | **p** | **All** | **Unspecific pattern** | **DKL diseases pattern** | **Complex pattern** | **p** |
| n | 633 | 447 | 97 | 89 |  | 198 | 97 | 54 | 47 |  |
| **EQ-5D mobility** | | |  |  | <0.001 |  |  |  |  | 0.001 |
| No problem | 323 (51.0) | 250 (55.9) | 49 (50.5) | 24 (27.0) |  | 151 (76.3) | 82 (84.5) | 44 (81.5) | 25 (53.2) |  |
| Some problems | 296 (46.8) | 191 (42.7) | 42 (43.3) | 63 (70.8) |  | 44 (22.2) | 14 (14.4) | 9 (16.7) | 21 (44.7) |  |
| Extreme problems | 14 (2.2) | 6 (1.3) | 6 (6.2) | 2 (2.2) |  | 3 (1.5) | 1 (1.0) | 1 (1.9) | 1 (2.1) |  |
| **EQ-5D self-care** | | |  |  | <0.001 |  |  |  |  | 0.009 |
| No problem | 507 (80.1) | 389 (87.0) | 64 (66.0) | 54 (60.7) |  | 173 (87.4) | 89 (91.8) | 50 (92.6) | 34 (72.3) |  |
| Some problems | 110 (17.4) | 54 (12.1) | 28 (28.9) | 28 (31.5) |  | 21 (10.6) | 6 (6.2) | 4 (7.4) | 11 (23.4) |  |
| Extreme problems | 16 (2.5) | 4 (0.9) | 5 (5.2) | 7 (7.9) |  | 4 (2.0) | 2 (2.1) | 0 (0.0) | 2 (4.3) |  |
| **EQ-5D usual activities** | | |  |  | <0.001 |  |  |  |  | 0.004 |
| No problem | 296 (46.8) | 221 (49.4) | 50 (51.5) | 25 (28.1) |  | 153 (77.3) | 81 (83.5) | 45 (83.3) | 27 (57.4) |  |
| Some problems | 301 (47.6) | 213 (47.7) | 37 (38.1) | 51 (57.3) |  | 39 (19.7) | 13 (13.4) | 9 (16.7) | 17 (36.2) |  |
| Extreme problems | 36 (5.7) | 13 (2.9) | 10 (10.3) | 13 (14.6) |  | 6 (3.0) | 3 (3.1) | 0 (0.0) | 3 (6.4) |  |
| **EQ-5D pain/discomfort** | | |  |  | <0.001 |  |  |  |  | 0.903 |
| No problem | 276 (43.6) | 196 (43.8) | 54 (55.7) | 26 (29.2) |  | 116 (58.6) | 56 (57.7) | 30 (55.6) | 30 (63.8) |  |
| Some problems | 313 (49.4) | 227 (50.8) | 40 (41.2) | 46 (51.7) |  | 75 (37.9) | 38 (39.2) | 22 (40.7) | 15 (31.9) |  |
| Extreme problems | 44 (7.0) | 24 (5.4) | 3 (3.1) | 17 (19.1) |  | 7 (3.5) | 3 (3.1) | 2 (3.7) | 2 (4.3) |  |
| **EQ-5D anxiety/depression** | | | |  | 0.124 |  |  |  |  | 0.775 |
| No problem | 309 (48.8) | 222 (49.7) | 51 (52.6) | 36 (40.4) |  | 105 (53.0) | 54 (55.7) | 30 (55.6) | 21 (44.7) |  |
| Some problems | 288 (45.5) | 202 (45.2) | 37 (38.1) | 49 (55.1) |  | 85 (42.9) | 39 (40.2) | 22 (40.7) | 24 (51.1) |  |
| Extreme problems | 36 (5.7) | 23 (5.1) | 9 (9.3) | 4 (4.5) |  | 8 (4.0) | 4 (4.1) | 2 (3.7) | 2 (4.3) |  |
| **EQ-5D VAS** | 65.0  [50.0, 80.0] | 70.0  [50.0, 80.0] | 65.0  [50.0, 80.0] | 60.0  [50.0, 70.0] | <0.001 | 77.50  [56.3, 90.0] | 80.0  [60.0, 90.0] | 72.50  [65.0, 88.5] | 70.0  [50.0, 86.0] | 0.143 |
| **EQ-5D score** | 0.79  [0.64, 0.89] | 0.80  [0.69, 0.89] | 0.81  [0.64, 0.91] | 0.71  [0.52, 0.83] | <0.001 | 0.90  [0.81, 1.0] | 0.91  [0.85, 1.0] | 0.89  [0.83, 1.0] | 0.84  [0.68, 1.0] | 0.073 |

*Notes.* Values are numbers (%) or median [interquartile range], as appropriate. *Abbreviations*: DKL, diabetes mellitus, chronic kidney and liver diseases; VAS, visual analogue scale.

**Supplementary Table 4. Linear regression for the association between the number and pattern of comorbidities and quality of life (Visual Analogue Scale and total EQ-5D score) of patients and caregivers of patients with atrial fibrillation**

|  | **β coefficient (95% CI)** | | | |
| --- | --- | --- | --- | --- |
|  | **N. comorbidities**  *(per each 1-disease increase)* | **Comorbidity pattern** | | |
|  |  |  | **Diabetes-kidney-liver** | **Complex** |
| **EQ-5D VAS** |  | |  |  |
| Patients | **-2.80 (-3.73 – -1.87)**  **p<0.001** |  | -2.77 (-7.17 – 1.63)  p=0.217 | **-9.81 (-14.40 – -5.22)**  **p<0.001** |
| Caregivers | **-1.29 (-2.55 – -0.02)**  **p=0.047** |  | 0.32 (-6.27 – 6.90)  p=0.924 | -6.03 (-13.13 – 1.07)  p=0.095 |
| **Total EQ-5D score** |  | |  |  |
| Patients | **-0.03 (-0.04 – -0.02)**  **p<0.001** |  | 0.00 (-0.06 – 0.06)  p=0.999 | **-0.10 (-0.16 – -0.04)**  **p**=**0.002** |
| Caregivers | **-0.02 (-0.03 – -0.004)**  **p=0.01** |  | 0.02 (-0.05 – 0.09)  p=0.649 | -0.07 (-0.15 – 0.002)  p=0.055 |

*Notes.* β coefficients are expressed relative to the Unspecific comorbidity pattern, which served as the reference category in all models. Models for the analysis on caregivers are adjusted for age, sex, education, caregiving time, formal/informal caregiver. Models for the analysis on patients are adjusted for age, sex, education, and living arrangements.
